# The Bic-C Family of Developmental Translational Regulators

**DOI:** 10.1155/2012/141386

**Published:** 2012-05-07

**Authors:** Chiara Gamberi, Paul Lasko

**Affiliations:** Department of Biology, McGill University, 3649 Promenade Sir William Osler, Montréal, QC, Canada H3G 0B1

## Abstract

Regulation of mRNA translation is especially important during cellular and developmental processes. Many evolutionarily conserved proteins act in the context of multiprotein complexes and modulate protein translation both at the spatial and the temporal levels. Among these, Bicaudal C constitutes a family of RNA binding proteins whose founding member was first identified in *Drosophila* and contains orthologs in vertebrates. We discuss recent advances towards understanding the functions of these proteins in the context of the cellular and developmental biology of many model organisms and their connection to human disease.

## 1. Introduction

Translational regulation of mRNA distributed asymmetrically in the early *Drosophila* embryo underlies pattern formation and germ cell specification. Furthermore, expression of certain proteins occurs only at definite stages of development. Exquisite, often partially redundant mechanisms of control ensure the coordination of the spatial and temporal expression of proteins with morphogenetic potential. These mechanisms have been reviewed recently [6]. Here we will discuss the case of one of such translational regulators, Bicaudal C (Bic-C), which is evolutionarily conserved, and for which there is recent accumulating functional evidence from both invertebrate and vertebrate model organisms suggesting that Bic-C is a fundamental regulator of cellular processes and an outstanding example of the fascinating complexity of the developmental mechanisms.

## 2. Materials and Methods

The sequences shown in this paper are listed in Table 1, and they were recovered by running BLAST [7] with the *Drosophila* sequence and the NCBI sequence database, using the Homologene feature at the NCBI. The sequences for the different* Drosophila *species were retrieved from FlyBase [8]. Sequences were aligned with Clustal W [1, 2].

## 3. Results and Discussion

### 3.1. *Bic-C*

The *Bic-C* gene was originally identified during a *Drosophila *screen for maternal genes affecting embryonic polarity [9]. In fact, adult females bearing *Bic-C* mutations in one of their second chromosomes produce embryos exhibiting anterior-posterior defects of severity ranging from anterior defects, to the development of bicaudal embryos composed of as few as four segments arranged as two, mirror-image posterior ends, to embryos that fail to cellularize [3]. This pleiotropy indicates that Bic-C participates in (or influences) many different pathways.

Early work demonstrated that Bic-C is required during oogenesis to establish anterior-posterior polarity in the oocyte [3, 5, 9, 10]. It encodes a 905-amino-acid (aa) RNA binding protein containing two canonical and three noncanonical KH RNA binding domains (KH2, 4 and KH 1, 3, 5, resp., aa 56–524) [3, 11, 12], a C-terminal Sterile Alpha Motif domain (SAM domain, aa 805–868, Prosite) [13], and a region rich in serine and glycine (aa 598–693). In the Bic-C protein, both the region containing the KH domains and the full-length, recombinant protein possess affinity for RNA [14, 15] with the full-length protein exhibiting more selective binding of synthetic probes* in vitro*. RNA binding is likely important to Bic-C function in fruit flies, as a spontaneous mutation (G296R) that affects the third KH domain, decreases RNA affinity *in vitro,* and exhibits a strong phenotype *in vivo *[3]. However, this mutation may be affecting more than RNA binding of the whole protein, for example, by perturbing secondary structure in its neighbourhood, as it may be the case for a similar mutation occurring in another KH domain [12]. If this were the case, the severity of the phenotype may be due to the combination of lack of RNA interaction and other defective pathways under Bic-C control in the wild type. The region containing the KH domains in two Bic-C orthologs shows conserved RNA binding capability in the mouse Bicc1 [16] and, surprisingly, not in the *C. elegans* GLD-3 [12].

**Figure 1:**
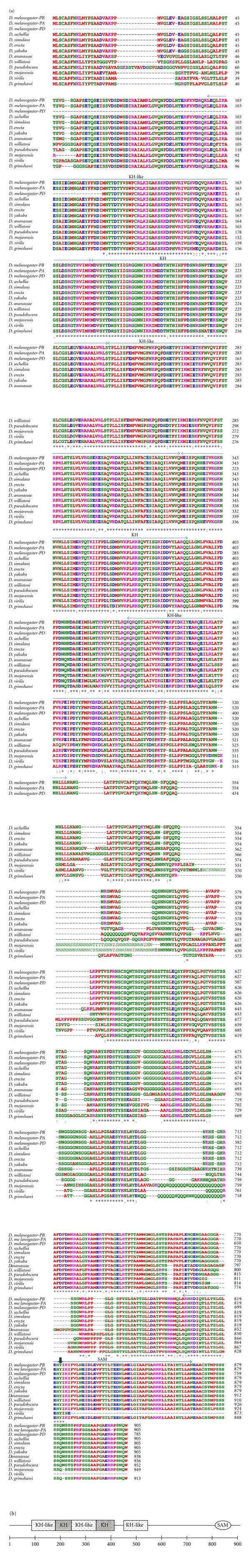
(a) Alignment of Bic-C sequences from 11 *Drosophila* species. Clustal W [1, 2] was used to align sequences extracted from FlyBase. Amino acid (aa) color coding is from Clustal W: red, small aliphatic, hydrophobic, and aromatics; blue, acidic; magenta, basic; green, hydroxyl, sulphydryl, amine, and glycine; grey, unusual aa. Symbols for aa conservation are from Clustal W: (asterisk ∗): positions with a single, fully conserved residue. (Colon  :): conservation between groups of strongly similar properties scoring >0.5 in the Gonnet PAM 250 matrix. (Period  .): conservation between groups of weakly similar properties scoring ≤0.5 in the Gonnet PAM 250 matrix. All three *D. melanogaster* Bic-C isoforms are shown (*PA*,* PB*, *PD*). The two canonical (KH) and three noncanonical (KH-like) KH RNA-binding modules are indicated (arrows, top). Domain assignment is as in [3] except for the fourth KH-related motif and the SAM domains, that are labelled according to the Pfam database [4]. A conserved, potentially phosphorylated, tyrosine is also indicated (arrowhead, top). Divergence occurs in regions of low complexity in the encoding DNA. Relative to the numbering of the *Drosophila* sequence: insertion at 555, variable length of the serine stretches around aa 623, and between aa 647–658 in the serine-glycine rich region. Further, after aa 715 there seems to be insertions of glutamine stretches of various lengths in *D. mojavensis*, *D. virilise*, and *D. grimshawi*. Finally, *D. ananassae* shows a short insertion at aa 770. The *D. virilise,* sequence results truncated. A TBLASTn search with the C-terminal region of Bic-C from *D. melanogaster* reveals many ESTs with similarity to the *D. melanogaster* sequence, suggesting a possible misannotation (not shown). Another region of possible sequencing misannotation in the *D. virilis* and the *D. mojavensis* Bic-C is italicized and not in bold type. Note that the *Bic-C* gene in *D. melanogaster* has nine mapped introns [5], and there is the possibility that the sequence was misannotated with this respect. (b) Block structure of the *D. melanogaster* Bic-C highlighting the protein motifs described in the text.

SAM domains are ancient modules present in most species that are commonly engaged in mediating protein-protein interaction [13, 17] and can multimerize [18, 19]. Multimerization of RNA binding proteins and RNA is most likely the basis for building RNP particles and a target of regulation. Interestingly, the SAM domain of the human BICC1 can form polymers *in vitro* [20] and some KH domains can mediate interactions between proteins [21, 22]. This is also the case for the C. elegans GLD-3 that interacts with the GLD-2 polymerase via its first KH domain [23] therefore it is likely that Bic-C is part of multiprotein complexes such as cellular RNPs. Certain SAM domains have also been implicated in RNA binding, as the case of *Drosophila* Smaug and *S. cerevisiae* Vts1 [24]. Interestingly, among all the *Drosophila *SAM domains, Bic-C contains the one most similar to Smaug's, which includes the critical residues for RNA interaction [25], suggesting the possibility that it may contribute to the Bic-C RNA binding capacity in the cell [17]. Studies of the vertebrate Bic-C homologs, whose targets are largely unknown, have suggested that presence of the SAM domain may mediate association with the P-bodies [26, 27]. Another interesting possibility is that the putative RNA binding and protein-protein interaction capabilities of the SAM domain may be regulated, possibly via posttranslational modifications. In this scenario protein modification in this domain may change the specificity and/or affinity of Bic-C for RNA to switch between protein and RNA binding activities in certain cellular or developmental contexts. Interestingly, a tyrosine residue in position 822 that can be phosphorylated in other SAM domains to regulate their activity is also conserved [28] (Figure 1).

### 3.2. Evolutionary Conservation of the Bic-C Protein

Bic-C is found in all the sequenced *Drosophila* species and its homologs are virtually identical to each other, except for regions of low complexity, where there are stretches of adjacent identical amino acids whose number varies in different species, the possible result of evolutionary mechanisms acting on triplet repeats or of stuttering sequencing polymerases (Figure 1).

**Figure 2:**
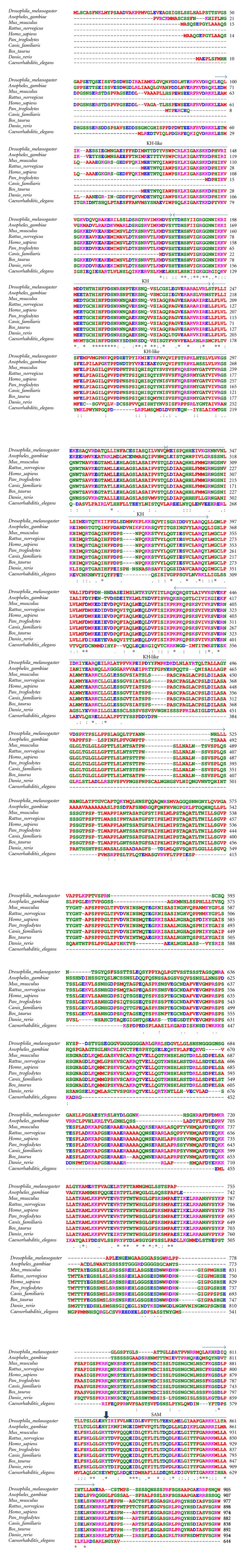
Bic-C orthologs. Clustal W [1, 2] was used to align sequences extracted from the NCBI sequence database. As in Figure 1, the two canonical (KH) and three noncanonical (KH-like) KH RNA-binding modules are indicated (arrows, top). Domain assignment is as in [3] except for the fourth KH-related motif and the SAM domains, that are labelled according to the Pfam database [4]. A conserved, potentially phosphorylated, tyrosine is also indicated (arrowhead, top). Amino acid (aa) color-coding is from Clustal W: red, small aliphatic, hydrophobic and aromatics; blue, acidic; magenta, basic; green, hydroxyl, sulphydryl, amine, and glycine; grey, unusual aa. Symbols for aa conservation are from Clustal W: (asterisk ∗): positions with a single, fully conserved residue. (Colon  :): conservation between groups of strongly similar properties-scoring >0.5 in the Gonnet PAM 250 matrix. (Period  .): conservation between groups of weakly similar properties-scoring ≤0.5 in the Gonnet PAM 250 matrix. Highlighted yellow: residues that contribute to RNA binding in the Smaug protein. Grey highlight denotes mild (versus strong) basic charges. Light blue highlights a charged aa in a conserved position, but an opposite electrical charge. The *Gallus gallus* genome also contains a predicted sequence with extensive homology to Bic-C (Table 1) and with a long extension at the N terminal end. Since there is no experimental evidence of the true starting methionine we did not include it in this alignment.

An alignment of Bic-C orthologs from different animals reveals extensive sequence conservation from aa 83 to 268, (referring to the *Drosophila* sequence). Between aa 269 and 303, the vertebrate proteins lack the acidic residues present in the two Dipterans (*D. melanogaster and Anopheles gambiae*) while the basic residues between aa 281 and 286 are conserved (Figure 2).

Similarly, between aa 417 and 423 the acidic residues are exchanged with a basic (K) or a neutral (G) residues, while the adjacent phenylalanine 424 is changed conservatively into a tyrosine, suggesting that the overall protein folding may be preserved and that the electrostatic environment may be different between the insect and the vertebrate proteins. Since this region contains possible KH-domain-like modules, this may influence their ability to interact with RNA by contributing positive charges that might help retain or stabilize the interaction with RNA. At aa 458, vertebrate sequences diverge from those of *Drosophila*, *Anopheles*, and *Caenorhabditis elegans*. These sequences show blocks of conservation (aa 712–737 and 815–863) interspersed with regions of divergence and one insertion of 38 residues at aa 778. The SAM domain is one such block of conservation, with its phosphorylatable tyrosine [28] that is invariant in all the sequences analysed and the identity (or conservative substitution) of most of the amino acids that contribute to create an environment conducive to RNA binding in the case of Smaug [24].

### 3.3. Bic-C and Translational Regulation

Evidence that Bic-C was involved in control of mRNA translation came first from studies in *Drosophila* where it was observed that Oskar, a well-studied morphogen, was upregulated in ovaries from Bic-C mutated females [14]. The identification of other mRNA targets coimmunoprecipitated with Bic-C yielded the *Bic-C* mRNA itself and several mRNAs encoding factors involved in the Wnt pathway, vesicular trafficking, and organization of the actin cytoskeleton [15]. Bic-C interacts directly with the Not3/5 subunit of the CCR4 deadenylase complex, and it is believed that, when bound to its target RNA, it is able to recruit the deadenylase. This shifts the cellular balance between polyadenylation and deadenylation towards the latter, impairing translation [15]. Since Not3/5 is also evolutionarily conserved, it is discussed below in the perspective of its contribution to the Bic-C complexes.

The other invertebrate family member for which there is substantial functional information is the *C. elegans* GLD-3. GLD-3 is involved in germline development and embryogenesis by regulating the time of expression of developmental factors [23, 29, 30]. GLD-3, via its first KH domain, interacts with GLD-2, a noncanonical polyA polymerase devoid of an RNA interaction domain of its own [23, 30]. Although it was expected that GLD-3 may tether GLD-2 to the RNA, a recent structural study could not find any RNA binding activity for the GLD-3 KH region [12]; therefore further studies are needed to elucidate how GLD-3 participates to *C. elegans* development.

In the *Drosophila* ovary Bic-C is present in cytoplasmic granules enriched for Trailer Hitch (Tral) and Me31B [31, 32], two proteins marking sponge bodies, ovarian organelles related to the repression of mRNA translation [33–35]. Mouse and *Xenopus* Bicc1 in cultured cells are also found within subcellular structures associated with mRNA silencing, the processing granules (P granules, [26, 27, 36]), strongly suggesting that the members of the Bic-C protein family may share a conserved function in translational control. For example, P bodies may destabilize mRNAs via the action of decapping enzymes such as Dcp1 in many tissues undergoing rapid mRNA turnover, while certain yeast mRNAs can be reversibly associated with P-bodies [37]. Further, in metazoans, deadenylation is often the rate-limiting, first step of mRNA decay [38]. While in the kidney, high turnover of certain mRNA may be instrumental to rapidly adapt organ function to the environmental changes, in tissues with a strong “anabolic” activity such as the ovary it would not be surprising to find that some maternal mRNAs are silenced and stored in cellular compartments refractory to translation during oogenesis, to be deployed later in the early embryo. Consistent with the possibility that Bic-C may not function by destabilizing its mRNA targets, no global changes in *Bic-C* mRNA stability were observed in the *Drosophila *ovary, neither by quantitative RT-PCR of ovarian total mRNA nor by *in situ* hybridization (Bic-C negatively regulates its own mRNA) [15]. While there seems to be a mild effect on stability of the polycystic kidney disease 2 (*Pkd2*) mRNA in the kidneys of the *Bicc1^−/−^* KO mice, in this case, no direct association of this mRNA with the Bicc1 protein was formally demonstrated [27]. It is also possible that only a fraction of the cellular Bic-C pool is involved in destabilization and degradation of mRNA targets, possibly constituting a distinct compartment. This scenario would have escaped detection via traditional biochemical methods because they cannot preserve the integrity of the tissues analyzed. Until more regulatory targets for the Bic-C family members will be identified, validated, and characterized functionally, this current puzzle will remain unanswered. 

### 3.4. Not3/5: An Evolutionarily Conserved Bic-C Partner Affecting mRNA Translation

Not3 is one of the subunits of the CCR4-NOT deadenylase, which is the predominant deadenylase, at least in the yeast *S. cerevisiae* [39–41]. Other subunits include CCR4, CAF1, NOT1-5 [40–44]. In *Drosophila* homologous genes are present for each of these subunits, with the exception of NOT3 and NOT5, for which there is only one gene displaying homology to both proteins [45]. Interestingly, Not3/5 does not contain any known protein domain, as identified via Prosite [46].

**Figure 3:**
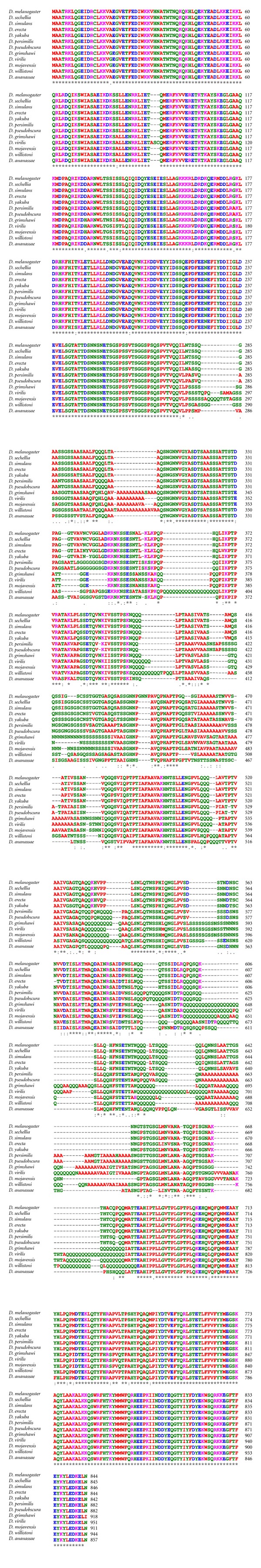
Not3/5 homologs from 12 *Drosophila* species. Clustal W [1, 2] was used to align sequences extracted from FlyBase. Amino acid (aa) color-coding is from Clustal W: red, small aliphatic, hydrophobic, and aromatics; blue, acidic; magenta, basic; green, hydroxyl, sulphydryl, amine, and glycine; grey, unusual aa. Symbols for aa conservation are from Clustal W: (asterisk ∗): positions with a single, fully conserved residue.  (Colon  :): conservation between groups of strongly similar properties-scoring >0.5 in the Gonnet PAM 250 matrix.  (Period  .): conservation between groups of weakly similar properties-scoring ≤0.5 in the Gonnet PAM 250 matrix.

**Figure 4:**
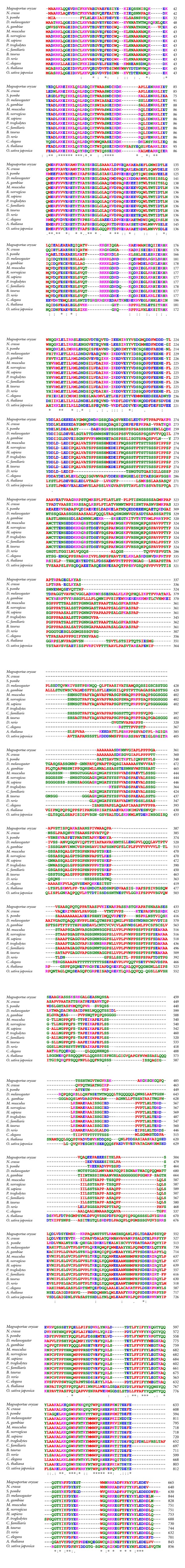
Not3/5 orthologs. Clustal W [1, 2] was used to align sequences extracted from the NCBI database. Amino acid (aa) color-coding is from Clustal W: red, small aliphatic, hydrophobic, and aromatics; blue, acidic; magenta, basic; green, hydroxyl, sulphydryl, amine, and glycine; grey, unusual aa. Symbols for aa conservation are from Clustal W: (asterisk ∗): positions with a single, fully conserved residue.  (Colon  :): conservation between groups of strongly similar properties-scoring >0.5 in the Gonnet PAM 250 matrix.  (Period  .): conservation between groups of weakly similar properties-scoring ≤0.5 in the Gonnet PAM 250 matrix.

*Drosophila* Not3/5 proteins are virtually identical in 12 species, the differences being concentrated in areas of low-sequence complexity (Figure 3). A BLAST search [7] reveals that besides insects and vertebrates, there are Not3/5 orthologs, in *fungi* (*S. cerevisiae*, *Schizosaccharomyces pombe*, as well as the mushrooms *Laccaria bicolor*, *Coprinopsis cinerea*, and *Schizophyllum commune*), vascular plants (e.g.,* Arabidopsis thaliana*, *Oryza sativa*, *Vitis vinifera*, and *Ustilago maydis*), and mosses (*Physcomitrella patens*). An alignment of complete sequences is shown in Figure 3. The conservation is highest at the N- and C-terminus of the protein (aa 1–238 and 680–844, with reference to the *Drosophila* sequence) where all the family members show extensive identity. Between aa 330 and 679 the sequences diverge with the orthologues from the two insects (*D. melanogaster* and *Anopheles gambiae*), the *fungi*, the higher eukaryotes, and the plants being more similar with each other than with members of a different group. Notably, the vertebrate sequences, with the exception of zebrafish that contains various small deletions, have blocks of almost complete identity in this region (Figure 4). The partial divergence in the central region of Not3/5 is likely due to the fact that the *Drosophila* gene is homologous to both the NOT3 and NOT5 genes and likely plays the functional roles of both yeast proteins, [45] a seemingly unique feature of *Drosophila [47]*. Not3/5 was recovered in a two-hybrid screen for proteins interacting with *Drosophila* Bic-C, and multiple pieces of evidence support the existence of this interaction *in vivo*: there is genetic interaction between *Bic-C* and *twin*, the *Drosophila* gene encoding for CCR4; other subunits of the CCR4-NOT complex can be coimmunoprecipitated with Bic-C from ovary extracts and the Bic-C target mRNAs that were tested were found with longer polyA tails in *Bic-C* mutants [15]. Although one study of vertebrate models could not detect differences in polyadenylation in a presumptive Bic-C target [27], due to the high homology of the Bic-C and NOT orthologs it is possible that Bic-C from other species can interact with NOT homologs and, possibly, other subunits of the deadenylase complex. These may contribute to the interaction only in the context of the assembled complex and may have therefore escaped detection in the *Drosophila* two-hybrid screen. Coimmunoprecipitation studies from tissue extracts and the precise mapping of the interaction domains on both proteins will be required to resolve this issue.

### 3.5. Multiple Bic-C Isoforms

*Drosophila* Bic-C has three predicted mRNA isoforms, RA, RB, and RD, that encode two identical (RA and RB) and one shorter (RD) proteins lacking the first 120 aa (Figure 1). These mRNA isoforms are expressed at different times during development (FlyBase): *Bic-C-RA* is expressed in the early embryo (0–6 hrs old) and in the adult female (i.e., most likely in the ovary), and *Bic-C-RB* is found mostly in late embryogenesis (7–22 hrs old). This is also consistent with our earlier report of multiple protein isoforms [14]. During the larval phases *Bic-C* is undetectable, and during pupation *Bic-C *expression is resumed, with its *RD* isoform being the most abundant and remaining prominent in adult males (FlyBase). The presence of two distinct mRNAs encoding the same amino acid sequence at definite developmental stages also suggests the possibility that they may be subjected to distinct regulation(s) in different tissues or at different developmental times and that the Bic-C activity may be required in specific time windows. This is consistent with a report that *Bic-C* function is especially needed at embryonic day (*E*) 18.5 during mouse development [27].

Interestingly, the mouse *Bicc1* gene and human *BICC1* also produce two distinct mRNAs by alternative splicing, which differ for the presence of exon 21 [26, 48] although no further functional information is known to date, so it is difficult to speculate if the presence of multiple Bic-C isoforms has conserved functional roles.

### 3.6. Bic-C and Polycystic Kidney Disease

In humans, two polycystic kidney disease (PKD) forms are caused by mutations in the *PKD1* and *PKD2* genes (autosomal, dominant [49–55]) or in PKHD1 (autosomal recessive, [52–55]). The link between Bic-C malfunction and PKD is compelling: two mouse models developing polycystic kidneys harbor mutations of the *Bicc1* gene [56]; Bic-C inactivation in *Xenopus* induces cystic kidneys [27, 57]; recently, a zebrafish model of PKD was validated that inhibits the Bicc1 function [58]. Finally, human studies on patients with renal disorders identified two mutations associated with the *BICC1* gene: one affecting the first KH domain and the other affecting the SAM domain [48], proving the relevance of the *Bic-C* animal models for understanding the etiology of this incurable disease.

In 3D cultures of mouse IMCD cells, depleting Bicc1 disrupts cadherin-mediated cell adhesion, normal epithelial polarization, proliferation, and apoptosis that prevent tubulomorphogenesis *in vitro *[59]. Interestingly, aspects of the *Drosophila* phenotype also affect cell migration and may influence cell-cell interaction and polarization. For example, migration of the follicle cells (FCs) in the ovary is defective in Bic-C mutant [3], resulting in eggs that remain open at the anterior end. This defect may occur because of inefficient communication between germ line and somatic cells, although to date we do not know the molecular pathway underlying this phenomenon (for an alternate possibility, see also Section 3.7).

In a recent paper [27] Tran and colleagues report that in a novel *Bicc1^−/−^* mutant mice and in *Xenopus* depleted for Bicc1 the *Pkd2* mRNA and its cognate protein are downregulated (29 and 54%, resp.), while both *Pkd1* and *Pdhd1* levels are unaffected. In the mouse these effects are clearest specifically at stage E18.5. The regulation appears to be mediated via a cellular microRNA, *miR-17* [27] that is also amplified in certain cancers [60]. Here Bicc1 may relieve the *miR-17*-mediated repression via a mechanism that does not involve regulation of the polyadenylation state of at least the mRNAs tested and may mildly impact mRNA stability [27]. The fact that the Bicc1 protein may bind multiple mRNAs and that it may be involved in the possible antagonistic regulation of the *miR-17* complexes, also assembled on multiple mRNAs, reinforces the view that the Bic-C orthologs are central to the regulation of many cellular processes and that many more aspects of their function await elucidation.

### 3.7. Other Bic-C Functions

Another hint to Bic-C function comes again from *Drosophila*, where the *Bic-C* mutants exhibit disrupted pattern of the cortical filamentous actin in the growing oocyte and abnormal actin-containing structures in the ooplasm that trap both the dorsal fate determinant Gurken [61–63] and other proteins that would normally be secreted [31, 32]. This function requires Trailer hitch, a protein originally identified in a screen for mutants for axial polarity that may regulate expression of endoplasmic reticulum (ER) exit site components on the ER surface. A malfunctioning secretory pathway could affect communication between the oocyte and the overlying FC and may affect their migration. Since many mRNAs involved in vesicular trafficking and/or organization of the actin cytoskeleton were also recovered in Bic-C immunoprecipitates [15], it is possible that their posttranscriptional control may contribute to the observed Bic-C defects. Lastly, and not mutually exclusive, the altered actin dynamics exhibited by the *Bic-C* and *Tral* mutants must also add to the observed inhibition of the normal dumping of nurse cell contents into the nascent oocyte during late oogenesis.

## 4. Concluding Remarks

Bic-C is an ancient protein conserved from *Drosophila* to man. Its mutation induces a pleiotropic phenotype. In fruit flies the Bic-C protein binds to RNAs involved in establishing the embryonic polarity, the Wnt pathway, actin dynamics and results in many observed defects, including abnormal development. In the vertebrates the better characterized aspect of lack of Bic-C function is the induction of cystic kidneys and the alteration of cell proliferation and three dimensional organization; however, defects in pancreatic and liver function and heterotaxia (i.e., randomization of the left-right symmetry) of the visceral organs have also been observed [26, 27]. Further, effects on the Wnt pathway have also been reported in human patients with renal displasia [48], as well as in mice and frogs [26]. Bicc1 is also expressed in the nervous system [58] which suggests that there may be novel aspects of its function ready to be discovered and that Bic-C homologs may be involved in fundamental, evolutionarily conserved mechanisms of determination of polarity, from establishment of the body axes to planar cell polarity.

The experimental evidence so far also suggests that Bic-C function may also be required at specific times of development in many species. Since Bic-C is a negative regulator of translation, we can expect at least part of the mutant phenotypes to be linked with inappropriate spatial and/or temporal regulation of gene expression. Further, Bic-C has multiple mRNA targets, and it exists in multiple isoforms in many organisms. At least in the case of one of the Bic-C interacting partners, the CCR4 deadenylase, it is proposed that multiple forms of this complex exist in higher vertebrates [47], as there are documented isoforms for a few of the complex subunits. Therefore, it is possible that the Bic-C-CCR4-dependent regulation acts via and is regulated by combinatorial mechanisms, with variant complexes having partially redundant function. This could also explain why all the individual molecular effects/phenotypes described for Bic-C tend to be mild and why years of concerted experimental efforts have yielded only a few proven targets for this gene, since many of the real targets would presumably not have been highly enriched compared to the controls.

## Figures and Tables

**Table 1:** Sequences used in this study.

Sequences	Species
Bic-C	
*Gene Bank ID *	
gi∣24584539	*D. melanogaster *B isoform
gi∣158300058	*A. gambiae*
gi∣13994223	*M. musculus*
gi∣109509376	*R. norvegicus*
gi∣122937472	*H. sapiens*
gi∣114631037	*P. troglodytes*
gi∣73953060	*C. familiaris*
gi∣194679417	*B. taurus*
gi∣292623098	*D. rerio*
gi∣212646112	*C. elegans*
gi∣118092391	*G. gallus*
*FlyBase ID *	
FBpp0080362	*D. melanogaster *B isoform
FBpp0080363	*D. melanogaster *D isoform
FBpp0080361	*D. melanogaster *A isoform
FBpp0118127	*D. ananassae*
FBpp0143734	*D. erecta*
FBpp0144300	*D. grimshawi*
FBpp0166588	*D. mojavensis*
FBpp0179414	*D. persimilis*
FBpp0287937	*D. pseudobscura*
FBpp0200128	*D. sechellia*
FBpp0222439	*D. simulans*
FBpp0232468	*D. virilis*
FBpp0253912	*D. willistoni*
FBpp0266309	*D. yakuba*

Not3/5	
*Gene Bank ID *	
gi∣39945962	*Magnaportae oryzae*
gi∣85075997	*Neurospora crassa*
gi∣19115701	*S. pombe*
gi∣19921660	*D. melanogaster*
gi∣158299738	*A. gambiae*
gi∣22122717	*M. musculus*
gi∣34854462	*R. norvegicus*
gi∣7657387	*H. sapiens*
gi∣114678945	*P. troglodytes*
gi∣73946891	*C. familiaris*
gi∣119911200	*B. taurus*
gi∣53933228	*D. rerio*
gi∣133901756	*C. elegans*
gi∣238481292	*A. thaliana*
gi∣115454389	*O. sativa japonica*
*FlyBase ID *	
FBpp0085398	*D. melanogaster*
FBpp0125948	*D. ananassae*
FBpp0129398	*D. erecta*
FBpp0147530	*D. grimshawi*
FBpp0160933	*D. mojavensis*
FBpp01852	*D. persimilis*
FBpp0288020	*D. pseudobscura*
FBpp0197981	*D. sechellia*
FBpp0208756	*D. simulans*
Bpp0227498	*D. virilis*
FBpp0243918	*D. willistoni*
FBpp0264455	*D. yakuba*

## References

[1] Larkin MA, Blackshields G, Brown NP (2007). Clustal W and Clustal X version 2.0. *Bioinformatics*.

[2] Goujon M, McWilliam H, Li W (2010). A new bioinformatics analysis tools framework at EMBL-EBI. *Nucleic Acids Research*.

[3] Mahone M, Saffman EE, Lasko PF (1995). Localized Bicaudal-C RNA encodes a protein containing a KH domain, the RNA binding motif of FMR1. *The Embo Journal*.

[4] Finn RD, Mistry J, Tate J (2010). The Pfam protein families database. *Nucleic Acids Research*.

[5] Ashburner M, Thompson P, Roote J (1990). The genetics of a small autosomal region of *Drosophila* melanogaster containing the structural gene for alcohol dehydrogenase—VII. Characterization of the region around the snail and cactus loci. *Genetics*.

[6] Lasko P (2011). Posttranscriptional regulation in *Drosophila* oocytes and early embryos. *Wiley Interdisciplinary Reviews: RNA*.

[7] Altschul SF, Madden TL, Schäffer AA (1997). Gapped BLAST and PSI-BLAST: a new generation of protein database search programs. *Nucleic Acids Research*.

[8] McQuilton P, St Pierre SE, Thurmond J (2012). FlyBase 101—the basics of navigating flyBase. *Nucleic Acids Research*.

[9] Mohler J, Wieschaus EF (1986). Dominant maternal-effect mutations of *Drosophila* melanogaster causing the production of double-abdomen embryos. *Genetics*.

[10] Schupbach T, Wieschaus E (1986). Germline autonomy of maternal-effect mutations altering the embryonic body pattern of *Drosophila*. *Developmental Biology*.

[11] Grishin NV (2001). KH domain: one motif, two folds. *Nucleic Acids Research*.

[12] Nakel K, Hartung SA, Bonneau F, Eckmann CR, Conti E (2010). Four KH domains of the C. elegans Bicaudal-C ortholog GLD-3 form a globular structural platform. *RNA*.

[13] Kim CA, Bowie JU (2003). SAM domains: uniform structure, diversity of function. *Trends in Biochemical Sciences*.

[14] Saffman EE, Styhler S, Rother K, Li W, Richard S, Lasko P (1998). Premature translation of *oskar* in oocytes lacking the RNA-binding protein Bicaudal-C. *Molecular and Cellular Biology*.

[15] Chicoine J, Benoit P, Gamberi C, Paliouras M, Simonelig M, Lasko P (2007). Bicaudal-C Recruits CCR4-NOT deadenylase to target mRNAs and regulates Oogenesis, Cytoskeletal organization, and its own expression. *Developmental Cell*.

[16] Bouvrette DJ, Price SJ, Bryda EC (2008). K homology domains of the mouse polycystic kidney disease-related protein, Bicaudal-C (Bicc1), mediate RNA binding *in vitro*. *Nephron Experimental Nephrology*.

[17] Lunde BM, Moore C, Varani G (2007). RNA-binding proteins: modular design for efficient function. *Nature Reviews*.

[18] Peterson AJ, Kyba M, Bornemann D, Morgan K, Brock HW, Simon J (1997). A domain shared by the Polycomb group proteins Scm and ph mediates heterotypic and homotypic interactions. *Molecular and Cellular Biology*.

[19] Qiao F, Bowie JU (2005). The many faces of SAM. *Science’s STKE*.

[20] Knight MJ, Leettola C, Gingery M, Li H, Bowie JU (2011). A human sterile alpha motif domain polymerizome. *Protein Science*.

[21] Chen T, Damaj BB, Herrera C, Lasko P, Richard S (1997). Self-association of the single-KH-domain family members Sam68, GRP33, GLD-1, and Qk1: role of the KH domain. *Molecular and Cellular Biology*.

[22] Di Fruscio M, Chen T, Bonyadi S, Lasko P, Richard S (1998). The identification of two *Drosophila* K homology domain proteins: KEP1 and SAM are members of the Sam68 family of GSG domain proteins. *Journal of Biological Chemistry*.

[23] Eckmann CR, Crittenden SL, Suh N, Kimble J (2004). GLD-3 and control of the mitosis/meiosis decision in the germline of *Caenorhabditis elegans*. *Genetics*.

[24] Aviv T, Lin Z, Lau S, Rendl LM, Sicheri F, Smibert CA (2003). The RNA-binding SAM domain of Smaug defines a new family of post-transcriptional regulators. *Nature Structural Biology*.

[25] Gamberi C, Johnstone O, Lasko P (2006). *Drosophila* RNA binding proteins. *International Review of Cytology*.

[26] Maisonneuve C, Guilleret I, Vick P (2009). Bicaudal C, a novel regulator of Dvl signaling abutting RNA-processing bodies, controls cilia orientation and leftward flow. *Development*.

[27] Tran U, Zakin L, Schweickert A (2010). The RNA-binding protein bicaudal C regulates *polycystin* 2 in the kidney by antagonizing miR-17 activity. *Development*.

[28] Schultz J, Ponting CP, Hofmann K, Bork P (1997). SAM as a protein interaction domain involved in developmental regulation. *Protein Science*.

[29] Eckmann CR, Kraemer B, Wickens M, Kimble J (2002). GLD-3, a bicaudal-C homolog that inhibits FBF to control germline sex determination in *C. elegans*. *Developmental Cell*.

[30] Wang L, Eckmann CR, Kadyk LC, Wickens M, Kimble J (2002). A regulatory cytoplasmic poly(A) polymerase in *Caenorhabditis elegans*. *Nature*.

[31] Kugler JM, Chicoine J, Lasko P (2009). Bicaudal-C associates with a trailer hitch/Me31B complex and is required for efficient gurken secretion. *Developmental Biology*.

[32] Snee MJ, Macdonald PM (2009). Bicaudal C and trailer hitch have similar roles in *gurken* mRNA localization and cytoskeletal organization. *Developmental Biology*.

[33] Jaglarz MK, Kloc M, Jankowska W, Szymanska B, Bilinski SM (2011). Nuage morphogenesis becomes more complex: two translocation pathways and two forms of nuage coexist in *Drosophila* germline syncytia. *Cell and Tissue Research*.

[34] Snee MJ, Macdonald PM (2009). Dynamic organization and plasticity of sponge bodies. *Developmental Dynamics*.

[35] Wilsch-Bräuninger M, Schwarz H, Nüsslein-Volhard C (1997). A sponge-like structure involved in the association and transport of maternal products during *Drosophila* oogenesis. *Journal of Cell Biology*.

[36] Olszewska M, Bujarski JJ, Kurpisz M (2012). P-bodies and their functions during mRNA cell cycle: mini-review. *Cell Biochemistry and Function*.

[37] Parker R, Sheth U (2007). P bodies and the control of mRNA translation and degradation. *Molecular Cell*.

[38] Chen CY, Shyu AB (1995). AU-rich elements: characterization and importance in mRNA degradation. *Trends in Biochemical Sciences*.

[39] Tucker M, Staples RR, Valencia-Sanchez MA, Muhlrad D, Parker R (2002). Ccr4p is the catalytic subunit of a Ccr4p/Pop2p/Notp mRNA deadenylase complex in Saccharomyces cerevisiae. *The Embo Journal*.

[40] Tucker M, Valencia-Sanchez MA, Staples RR, Chen J, Denis CL, Parker R (2001). The transcription factor associated Ccr4 and Caf1 proteins are components of the major cytoplasmic mRNA deadenylase in *Saccharomyces cerevisiae*. *Cell*.

[41] Chen J, Chiang YC, Denis CL (2002). CCR4, a 3′-5′ poly(A) RNA and ssDNA exonuclease, is the catalytic component of the cytoplasmic deadenylase. *The Embo Journal*.

[42] Thore S, Mauxion F, Séraphin B, Suck D (2003). X-ray structure and activity of the yeast Pop2 protein: A nuclease subunit of the mRNA deadenylase complex. *EMBO Reports*.

[43] Daugeron MC, Mauxion F, Séraphin B (2001). The yeast POP2 gene encodes a nuclease involved in mRNA deadenylation. *Nucleic Acids Research*.

[44] Chen J, Rappsilber J, Chiang YC, Russell P, Mann M, Denis CL (2001). Purification and characterization of the 1.0 MDa CCR4-NOT complex identifies two novel components of the complex. *Journal of Molecular Biology*.

[45] Oberholzer U, Collart MA (1998). Characterization of NOT5 that encodes a new component of the Not protein complex. *Gene*.

[46] Sigrist CJA, Cerutti L, de Castro E (2010). PROSITE, a protein domain database for functional characterization and annotation. *Nucleic Acids Research*.

[47] Temme C, Zaessinger S, Meyer S, Simonelig M, Wahle E (2004). A complex containing the CCR4 and CAF1 proteins is involved in mRNA deadenylation in *Drosophila*. *The Embo Journal*.

[48] Kraus MR, Clauin S, Pfister Y (2012). Two mutations in human BICC1 resulting in Wnt pathway hyperactivity associated with cystic renal dysplasia. *Human Mutation*.

[49] Burn TC, Connors TD, Dackowski WR (1995). Analysis of the genomic sequence for the autosomal dominant polycystic kidney disease (PKD1) gene predicts the presence of a leucine-rich repeat: the American PKD1 consortium (APKD1 Consortium). *Human Molecular Genetics*.

[50] Hughes J, Ward CJ, Peral B (1995). *The polycystic kidney disease* 1 (PKD1) gene encodes a novel protein with multiple cell recognition domains. *Nature Genetics*.

[51] Mochizuki T, Wu G, Hayashi T (1996). PKD2, a gene for polycystic kidney disease that encodes an integral membrane protein. *Science*.

[52] Hildebrandt F, Otto E, Rensing C (1997). A novel gene encoding an SH3 domain protein is mutated in nephronophthisis type 1. *Nature Genetics*.

[53] Onuchic LF, Furu L, Nagasawa Y (2002). *PKHD1*, the polycystic kidney and hepatic disease 1 gene, encodes a novel large protein containing multiple immunoglobulin-like plexin-transcription-factor domains and parallel beta-helix 1 repeats. *American Journal of Human Genetics*.

[54] Ward CJ, Hogan MC, Rossetti S (2002). The gene mutated in autosomal recessive polycystic kidney disease encodes a large, receptor-like protein. *Nature Genetics*.

[55] Xiong H, Chen Y, Yi Y (2002). A novel gene encoding a TIG multiple domain protein is a positional candidate for autosomal recessive polycystic kidney disease. *Genomics*.

[56] Cogswell C, Price SJ, Hou X, Guay-Woodford LM, Flaherty L, Bryda EC (2003). Positional cloning of jcpk/bpk locus of the mouse. *Mammalian Genome*.

[57] Tran U, Pickney LM, Özpolat BD, Wessely O (2007). Xenopus bicaudal-C is required for the differentiation of the amphibian pronephros. *Developmental Biology*.

[58] Bouvrette DJ, Sittaramane V, Heidel JR, Chandrasekhar A, Bryda EC (2010). Knockdown of bicaudal C in zebrafish (*Danio rerio*) causes cystic kidneys: a nonmammalian model of polycystic kidney disease. *Comparative Medicine*.

[59] Fu Y, Kim I, Lian P (2010). Loss of Bicc1 impairs tubulomorphogenesis of cultured IMCD cells by disrupting E-cadherin-based cell-cell adhesion. *European Journal of Cell Biology*.

[60] Jovanovic M, Hengartner MO (2006). miRNAs and apoptosis: RNAs to die for. *Oncogene*.

[61] Neuman-Silberberg FS, Schupbach T (1993). The *Drosophila* dorsoventral patterning gene gurken produces a dorsally localized RNA and encodes a TGF*α*-like protein. *Cell*.

[62] Ray RP, Schüpbach T (1996). Intercellular signaling and the polarization of body axes during *Drosophila* oogenesis. *Genes & Development*.

[63] Wilhelm JE, Buszczak M, Sayles S (2005). Efficient protein trafficking requires trailer hitch, a component of a ribonucleoprotein complex localized to the ER in *Drosophila*. *Developmental Cell*.

